# Computational Model of the Effective Thermal Conductivity of a Bundle of Round Steel Bars

**DOI:** 10.3390/ma18020373

**Published:** 2025-01-15

**Authors:** Rafał Wyczółkowski, Marek Gała

**Affiliations:** 1 Department of Production Management, Czestochowa University of Technology, Armii Krajowej 19, 42-200 Czestochowa, Poland; 2 Institute of Electric Power Engineering, Czestochowa University of Technology, Armii Krajowej 17, 42-200 Czestochowa, Poland; marek.gala@pcz.pl

**Keywords:** heat treatment, steel bars, bar bundle, effective thermal conductivity, thermal resistance, unit cell

## Abstract

During the heat treatment of round steel bars, a heated charge in the form of a cylindrically formed bundle is placed in a furnace. This type of charge is a porous granular medium in which a complex heat flow occurs during heating. The following heat transfer mechanisms occur simultaneously in this medium: conduction in bars, conduction within the gas, thermal radiation between the surfaces of the bars, and contact conduction across the joints between the adjacent bars. This complex heat transfer can be quantified in terms of effective thermal conductivity. This article presents the original model of the effective thermal conductivity of a bundle of round steel bars. This model is based on the thermo-electric analogy. Each heat transfer mechanism is assigned an appropriate thermal resistance. As a result of the calculations, the impact of the following parameters on the intensity of heat transfer in the bundle was examined: temperature, the thermal conductivity of the bars, the thermal conductivity of the gas, diameter, and emissivity of the bar, and bundle porosity. Calculations were performed for a temperature range of 25–800 °C, covering a wide spectrum of variables, including bar diameters, bundle porosity, and type of gas. The knowledge obtained thanks to the calculations performed will facilitate the optimization of heat treatment processes for the considered charge. The greatest scientific value of the presented research is the demonstration that, thanks to the developed computational model, it is possible to analyze a very complex heat transfer phenomenon using relatively simple mathematical relationships.

## 1. Introduction

**Figure 1:**
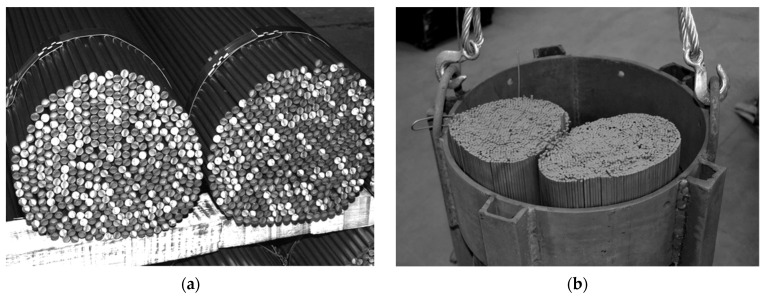
Bundles of bars as an example of a steel porous charge: (**a**) view of the bundle depicting its heterogeneous structure; (**b**) bundles prepared for heat treatment in a soaking furnace.

Amidst the climate crisis marked by global warming, numerous industries are pursuing technological advancements to lower energy usage and cut greenhouse gas emissions [1,2,3,4,5,6]. This issue also pertains to the metallurgical sector involved in the heat treatment of steel products [7,8,9,10,11]. For this reason, continuous research focuses on exploring different aspects of the heat treatment processes for steel products [12,13,14,15,16,17]. Efforts to improve the efficiency of heat treatment processes include the development of algorithms that enable the most accurate prediction of the current temperature of the heated element. This problem is particularly complex when the treated metal has a porous structure [18,19,20,21,22]. One example of such a charge are the cylindrical bundles of steel bars [23]. As illustrated in Figure 1, the analyzed elements feature gaps filled with gas and lack continuity of the solid phase in the radial direction. These specific characteristics significantly affect the heating process of the bundle. In such a medium, thermal energy is transferred through a combination of: (I) conduction within individual bars, (II) conduction through the gas, (III) contact conduction between adjacent bars, and (IV) thermal radiation between bar surfaces. The complexity of these mechanisms makes it difficult to create an exact mathematical model for the heating process of a bundle without accounting for many intricate relationships. However, this problem can be greatly reduced by introducing the concept of effective thermal conductivity (ETC or *k_ef_*) This parameter is widely applied in the study of porous media [24,25,26,27]. The use of effective thermal conductivity enables the quantitative characterization of transient heat transfer within a bar bundle, facilitating the determination of its heating time [23,28,29].

The most reliable way to determine the ETC is through experimental investigations. The steady-state method with a guarded hot plate apparatus is commonly used to measure the effective thermal conductivity of cellular and porous materials [30,31,32,33]. This method, however, demands specialized equipment, is time-intensive, and requires the preparation of specific test samples. A significant drawback of these measurements is their limited generalizability, as the results are specific to the tested material samples. Consequently, model calculations are frequently employed as a practical alternative for determining the ETC of porous materials [34].

Numerous analytical models for estimating the effective thermal conductivity of two-phase media (solid–gas) are available in the literature. The models used are generally divided into two groups. The first group consists of simple models, while the second group comprises complex models. The simple models, also referred to as “rigid”, express ETC solely based on the thermal conductivities of the solid phase (*k*_s_) and gas phase (*k_g_*), as well as the medium’s porosity (*φ*). The most commonly used simple models include structural models such as the parallel model, series model, Effective Medium Theory (EMT), and the Maxwell–Eucken model [35]. The complex models, also referred to as “flexible”, consider additional (secondary) parameters, such as thermal contact resistance (TCR) or thermal contact conductance (TCC), heat transfer through radiation, and the mean size of grains or voids [36]. Some of the more popular complex models are: the Krischer model (which is the weighted harmonic average of the parallel and series models) [35], Kunii–Smith [37], and Zehner–Schlünder [38]. As shown, the models mentioned above, both simple and complex are not appropriate for accurately determining the effective thermal conductivity of bar bundles [39]. This is due to the fact that these models do not account for the specific heat transfer processes that occur during the heating of the discussed charge. This particularly pertains to two phenomena: contact conduction and thermal radiation.

This article presents the original model of the effective thermal conductivity of a bundle of round steel bars. This model takes into account the following modes of heat transfer: conduction in the bars, conduction in the gas, contact conduction at the junctions of the bars, and thermal radiation between the surfaces of adjacent bars. The heat transfer analysis does not consider gas convection. As the experimental tests have shown, due to the small dimensions of the voids between the bars, this phenomenon can be neglected [40]. This is an analytical–empirical model because the function describing contact conduction occurring in its structure was determined on the basis of experimental research [41]. The greatest scientific value of the presented research is the demonstration that thanks to the developed mathematical model, it is possible to analyze a very complex heat transfer phenomenon using relatively simple mathematical relationships.

## 2. Materials and Methods

The presented model is based on the analysis of thermal resistances relating to individual heat transfer mechanisms that occur during the heating of the medium considered. This approach is based on the analogy of the two phenomena, namely electrical and thermal conduction, which results from the similarity of the mathematical description of Ohm and Fourier laws [42]. In numerous instances, this method serves as an efficient alternative for tackling intricate heat transfer issues in heterogeneous systems, providing a simpler solution compared to more advanced numerical techniques [43,44,45,46].

**Figure 2:**
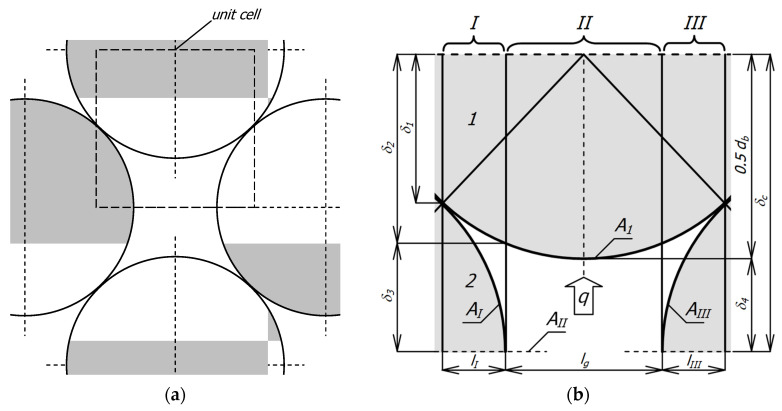
Geometric model of the considered medium: (**a**) a fragment of flatbed of round bars with a staggered arrangement; (**b**) a unit cell.

The basis for deriving the appropriate mathematical relationships is the geometric model of the medium. In this case, it is a layered, flat bed of round bars with a staggered arrangement, a fragment of which is shown in Figure 2a. Then, a periodically repeating element is isolated in this system, called a unit cell (Figure 2b). A unit cell approach is commonly used in heat transfer modeling in porous media [27,47]. This cell contains parts of bars from two adjacent layers—the upper layer was marked with the number *1*, and the lower layer with the number *2*. The free space (gap) between the bars is filled with gas. The cell is divided into three sections *I*–*III* (due to symmetry, sections *I* and *III* are the same). Thanks to such division, eight elements are distinguished in the cell. In sections *I* and *III* there are six elements (*E_I1_*, *E_Ig_*, *E_I2_*, *E_III1_*, *E_III2_*, and *E_IIIg_*) corresponding to the part of the bars from the upper layer (*E_I1_*, *E_III1_*) the part of gap (*E_Ig_*, *E_IIIg_*) and the part of the bars from the lower layer (*E_I2_*, *E_III2_*). In section *II* there are two elements relating to the part of the bar from the upper layer *E_II1_* and the remaining part of the gap *E_IIg_*.

The arrangement shown in Figure 2a represents the most general case of the considered charge when there is a gap between the bars—its width is determined by the value of the parameter *x_g_* from Equation (3). A special case is when *x_g_* = 0, in such a situation, the considered bed is closely packed (with minimal porosity). A charge with such porosity can be found in industrial practice, but it requires extraordinary care when arranging the bars. In most cases, heat-treated bar bundles are characterized by a lack of maximal packing—as shown in Figure 1a. Therefore, the system presented in Figure 2a is the most adequate for the analyzed issue.

It is assumed that a one-dimensional, steady heat flow takes place within the cell characterized by the heat flux vector *q*. The direction of the *q* vector is parallel to the planes of division of the cell into sections.

Two parameters are set to define the geometry of the system: the bar diameter *d_b_* and the distance between the bars in layer *l_g_*, which is also the width of section *II*. These values determine the height of cell *δ_c_* and the widths of sections *I* and *III*:

(1) $$\delta_{c}=\sqrt{{d_{b}}^{2}-{\left(0.5\left(d_{b}+l_{g}\right)\right)}^{2}},$$

(2) $$l_{I}=l_{III}=0.25\left(d_{b}-l_{g}\right).$$

The parameter *l_g_* is defined as a fraction of the bar diameter:

(3) $$l_{g}=x_{g}\cdot d_{b}.$$

The next quantity describing the system’s geometry is the length of the bars *l_b_*. Since the phenomenon is considered one-dimensional, a unit value was adopted for this parameter (*l_b_* = 1 m). Next, the relative heat transfer surface areas in individual sections are defined *f_I_*, *f_II_,* and *f_III_*:

(4) $$f_{I}=f_{III}=\frac{A_{I}}{A_{I}+A_{II}+A_{III}}=\frac{l_{I}}{2l_{I}+l_{II}},$$

(5) $$f_{II}=\frac{A_{II}}{A_{I}+A_{II}+A_{III}}=\frac{l_{II}}{2l_{I}+l_{II}},$$

where

(6) $$A_{I}=A_{III}=l_{b}\cdot l_{I},$$

(7) $$A_{II}=l_{b}\cdot l_{II}.$$

where *A_I_*–*A_III_* are the surface areas of individual sections.

The value of ETC for the considered cell is calculated using the definition of thermal resistance to conduction through the plane wall [48]:

(8) $$ETC=\frac{\delta_{c}}{R_{to}},$$

where *R_to_* and *δ_c_* are the total thermal resistance and the height of the cell. Due to the adopted cell division, the resistance *R_to_* is calculated as the parallel combination of resistances of individual sections:

(9) $$R_{to}={\left(\frac{1}{R_{I}}+\frac{1}{R_{II}}+\frac{1}{R_{III}}\right)}^{-1}={\left(\frac{2}{R_{I}}+\frac{1}{R_{II}}\right)}^{-1}$$

The resistance *R_I_* depends on the five resistances: conduction within the bars (*R_I1_* and *R_I2_*), conduction in the gas *R_Ig_*, contact conduction *R_ct_*, and radiation *R_Ird_*. Meanwhile, the resistance *R_II_* is a function of three resistances: conduction within the bar *R_II1_*, conduction in the gas *R_IIg,_* and radiation *R_IIrd_*. The resistances *R_I_* and *R_II_* are calculated using the following relationships:

(10) $$R_{I}=R_{I1}+{\left(\frac{1}{R_{Ig}}+\frac{1}{R_{Ird}}+\frac{1}{R_{ct}}\right)}^{-1}+R_{I2},$$

(11) $$R_{II}=R_{II1}+{\left(\frac{1}{R_{IIg}}+\frac{1}{R_{IIrd}}\right)}^{-1}.$$

If heat conduction in section *I* concerns a flat layer with dimensions *l_x_* and thermal conductivity *k_x_*, then its conduction resistance would be described by the relationship:

(12) $$R_{cd}=\frac{l_{x}}{f_{I}\cdot k_{x}}.$$

However, the conduction resistances (*R*_*I*1_, *R*_*I*2_, *R_Ig_*, *R*_*II*1_, *R_IIg_*) appearing in Equations (10) and (11) pertain to elements with varying heights and, therefore, cannot be calculated using relationship (12). The values of these resistances are calculated according to a particular procedure, which is described in detail in the work [49]. The minimum and maximum dimensions of each element of the elementary cell are listed in Table 1 and are described by the following relationships:

(13) $$\delta_{1}=\sqrt{{\left(0.5d_{b}\right)}^{2}-{\left(0.25\left(d_{b}+l_{g}\right)\right)}^{2}},$$

(14) $$\delta_{2}=\sqrt{{\left(0.5d_{b}\right)}^{2}-{\left(0.5l_{g}\right)}^{2}},$$

(15) $$\delta_{3}=\delta_{c}-\delta_{2},$$

(16) $$\delta_{4}=\delta_{c}-0.5d_{b}.$$

Thermal contact resistance *R_ct_*, occurring in sections *I* and *III*, describes the intensity of heat transfer through the contact areas between the bars of the individual layers of the bed. The resistance *R_ct_* about one section (due to the symmetry of the cell) is twice the total contact thermal resistance *R_tct_*:

(17) $$R_{ct}=2R_{tct}.$$

The resistance *R_tct_* is the reciprocal of the thermal contact conductance *h_ct_* [50,51,52]:

(18) $$R_{tct}=\frac{1}{h_{ct}}.$$

The authors in previous work [41] analyzed the issue of contact conduction in beds of round bars. It was established that for a bed of bars with a staggered arrangement, changes in the coefficient *h_ct_* as a function of bar diameter *d_b_* and bed temperature *t* could be described by the following relationship:

(19) $$h_{ct}=-2.21d_{b}+176.7+0.156t+\left(0.0057d_{b}-2.1\right)\cdot 10^{-4}t^{2}.$$

Therefore:

(20) $$R_{ct}={\left(0.5\left(-2.21d_{b}+176.7+0.156t+\left(0.0057d_{b}-2.1\right)\cdot 10^{-4}t^{2}\right)\right)}^{-1}.$$

Radiation resistances *R_Ird_* and *R_IIrd_* are described by formulas:

(21) $$R_{Ird}=\frac{R_{rd}}{f_{I}},$$

(22) $$R_{IIrd}=\frac{R_{rd}}{f_{II}}.$$

Resistance *R_rd_* is described by the following equation [53]:

(23) $$R_{rd}=\frac{X_{rd}}{4\sigma_{c}T_{m}^{3}},$$

where *σ_c_* is the Stefan–Boltzmann constant, *T_m_* is the mean absolute temperature of the considered medium, and *X_rd_* is a coefficient, whose value depends on the emissivity of the bars *ε_b_*, the shape, and the relative orientation of the surfaces limiting the area of radiative heat transfer within the elementary cell. In the analyzed case, this space is confined by fragments of the surfaces of three bars designated as *A_I_*, *A_III,_* and *A_1_*. Since these surfaces do not entirely enclose the radiative heat transfer space, an apparent surface *A_ap_* is introduced in place of the gap surface *A_II_*. This is a standard procedure for analyzing radiative heat transfer in open systems [54]. Since the surface *A_ap_* does not reflect radiation, it possesses black body properties (*ε_ap_* = 1). The following relationships describe the areas of individual surfaces:

(24) $$A_{I}=A_{III}=\frac{3.14d_{b}\;arc\;\cos\;\left(0.5\left(d_{b}+l_{g}\right)d_{b}^{-1}\right)}{360{}^{\circ}}\cdot l_{b},$$

(25) $$A_{1}=\left(1.57d_{b}-2A_{I}\right)\cdot l_{b},$$

(26) $$A_{ap}=l_{g}\cdot l_{b}.$$

Next, the three surfaces from the bottom layer (two convex surfaces *A_I_*, *A_III_*, and the flat surface *A_ap_*) are treated as one concave surface *A*_2_:

(27) $$A_{2}=A_{I}+A_{ap}+A_{III}=2A_{I}+A_{ap}.$$

The surface *A*_2_ is assigned an equivalent emissivity *ε_eq_*, which is determined as the weighted average of the emissivities of the *A_I_* and *A_ap_* surfaces:

(28) $$\varepsilon_{eq}=\frac{2A_{I}}{A_{2}}\varepsilon_{b}+\frac{A_{ap}}{A_{2}}\varepsilon_{ap}.$$

Therefore, radiative heat transfer in the considered cell is reduced to the exchange of radiation between two enclosed surfaces (convex *A*_1_ and concave *A*_2_). The effective emissivity of such a system is described by the equation [54]:

(29) $$\varepsilon_{ef}={\left(\frac{1}{\varepsilon_{b}}+\frac{A_{1}}{A_{2}}\left(\frac{1}{\varepsilon_{eq}}-1\right)\right)}^{-1}.$$

Finally, the coefficient *X_rd_* from Equation (23) is described by the relationship:

(30) $$X_{rd}=\frac{2l_{I}+l_{g}}{\left(0.5\pi d_{b}-2l_{I}\right)\;\varepsilon_{ef}}.$$

**Figure 3:**
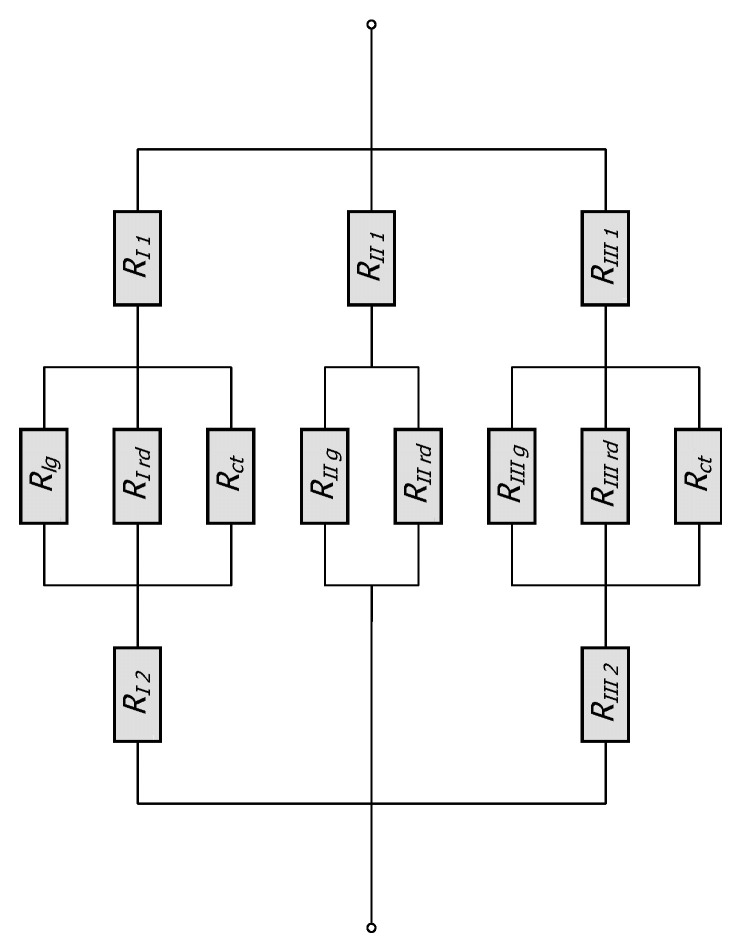
Thermal resistance networks for the considered unit cell.

Therefore, the total thermal resistance of the elementary cell *R_to_* is calculated as a combination of series and parallel connections of thirteen distinct thermal resistances related to: conduction within the bars (five resistances), conduction in the gas area (three resistances), contact conduction (two resistances), and thermal radiation (three resistances). The equivalent network of these resistances is shown in Figure 3.

## 3. Results and Discussion

In the model calculations, the following parameters were the input data:

- bar diameter *d_b_*;
- distance between the bars *l_g_*;
- thermal conductivity of steel *k_s_*;
- thermal conductivity of gas *k_g_*;
- thermal contact conductance *h_ct_*;
- bar surface emissivity *ε_b_*.

All calculations were performed for the temperature range 25–800 °C. The maximum value of this range is related to the annealing temperature of carbon steels. This parameter depends on the type of annealing and the carbon content in the steel. For instance, in stress-relief annealing, the temperature range is 500–650 °C, while for soft annealing, it is 680–750 °C [55]. Due to the large width of this range, it was assumed that the coefficients *h_ct_*, *k_s_*, and *k_g_* vary with temperature. The changes in *h_ct_* are described by Equation (19). Changes in the thermal conductivity of steel are described by the following equation [56]:

(31) $$\begin{array}{c} k_{s}=112.51x_{c}^{2}-100.85x_{c}+68.89-\left(0.42x_{c}^{2}+0.318x_{c}+0.065\right)\;t\\ +\left(334.05x_{c}^{2}-247.27x_{c}+18.59\right)\cdot 10^{-6}t^{2} \end{array},$$

where *t* is steel temperature expressed in degrees Celsius and *x_c_* is the percentage of carbon in the steel.

Calculations were made for twelve different bundle geometries, assuming four bar diameters (10, 20, 30, and 40 mm) and three distances between the bars.

First, calculations were made for bundles of steel bars with a carbon content of 0.2% heated in air, which corresponds to heat treatment in a furnace without a protective atmosphere. The changes in air thermal conductivity in the temperature range of 25–800 °C are described by the equation:

(32) $$k_{g-air}=-2.88\cdot 10^{-8}t^{2}+8.05\cdot 10^{-5}t+0.024.$$

Subsequently, calculations were also performed for the case when the gas filling the bundle voids is hydrogen. This gas as an atmosphere in heat treatment processes offers several benefits: helps prevent oxidation of metals and due to high thermal conductivity, enhances the uniformity of heating and cooling processes. These features can improve the overall quality of the treated materials. For this reason, hydrogen is often used in the annealing processes [57,58]. The thermal conductivity of hydrogen in the function of temperature was described by a linear equation:

(33) $$k_{g-hyd}=0.0004t+0.184.$$

Equations (32) and (33) were obtained by approximating tabulated data [59]. The values of the thermal conductivity of steel, air, and hydrogen for the temperatures 25 °C and 800 °C are listed in Table 2. It was also assumed that the emissivity of the bar surface is 0.7, which is a typical value for rolled steel products [60,61].

**Figure 4:**
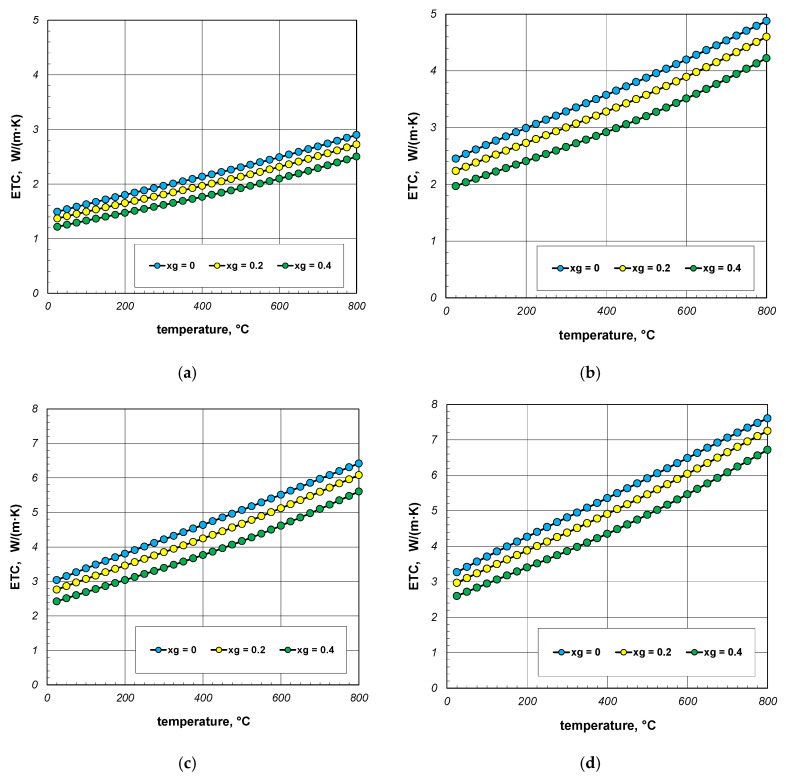
Effective thermal conductivity of the bar bundle depending on the temperature and porosity: (**a**) 10 mm bars, (**b**) 20 mm bars, (**c**) 30 mm bars, and (**d**) 40 mm bars.

The results of ETC calculations for the case when the gas filling the bundle is air, divided by individual bar diameters, are shown in Figure 4. Each graph shows the results for three charge porosities *φ* in the temperature function. Parameter *φ* was regulated by changing the gap width *l_g_* as a multiple of the bar diameter (Equation (3)). Calculations were performed for three values of the coefficient *x_g_*: 0; 0.2 and 0.4. The porosities of the bundle corresponding to the respective values of *x_g_* are listed in Table 3.

**Figure 5:**
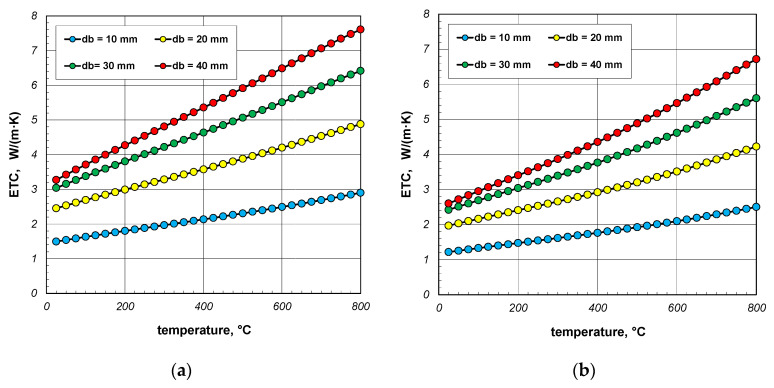
Effective thermal conductivity of the bar bundle depending on the temperature and bar diameter: (**a**) bundle porosity 0.09 (*x*_g_ = 0) and (**b**) bundle porosity 0.21 (*x*_g_ = 0.4).

**Figure 6:**
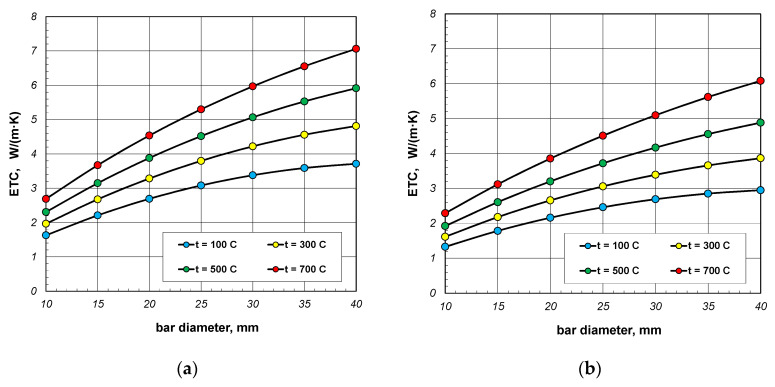
Effective thermal conductivity of the bar bundle depending on bar diameter and temperature: (**a**) bundle porosity 0.09 (*x*_g_ = 0) and (**b**) bundle porosity 0.21 (*x*_g_ = 0.4).

As can be seen from the above graphs, ETC in each case increases linearly as a function of temperature. Thermal radiation between the surfaces of the bars is primarily responsible for this tendency of changes. ETC also increases as the bar diameter increases. To better show this last effect, additional graphs were prepared in Figure 5 and Figure 6. The graphs in Figure 5 show the results obtained for bundles with porosity 0.09 and 0.21. Each graph compares the results for individual bar diameters. It can be seen that for both bundle porosities, the effect of the bar diameter on ETC changes is very similar. The results from Figure 4 and Figure 5 further show that increasing the charge porosity causes a decrease in ETC.

The results from Figure 6 show the changes in ETC as a function of bar diameter for four values of charge temperature: 100, 300, 500, and 700 °C. The lines in these charts are not straight. The changes in bundle ETC as a function of bar diameter can be described using a second-degree polynomial:

(34) $$ETC\left(d_{b}\right)=-A_{1}d_{b}^{2}+B_{1}d_{b}+C_{1},$$

where the constants *A*_1_, *B*_1,_ and *C*_1_ depend on the temperature and porosity of the charge.

An important problem when analyzing the heat transfer in a bar bundle is the grade to which the ETC value differs from the thermal conductivity of the bars, expressed by the *k_s_* coefficient. The difference between the values of these two parameters shows the extent to which the process of heat flow in the bundle differs from heat conduction in the steel itself. This discrepancy was shown using a parameter called reduced effective thermal conductivity RETC, which was defined as the product of:

(35) $$RETC=\frac{ETC}{k_{s}}.$$

**Figure 7:**
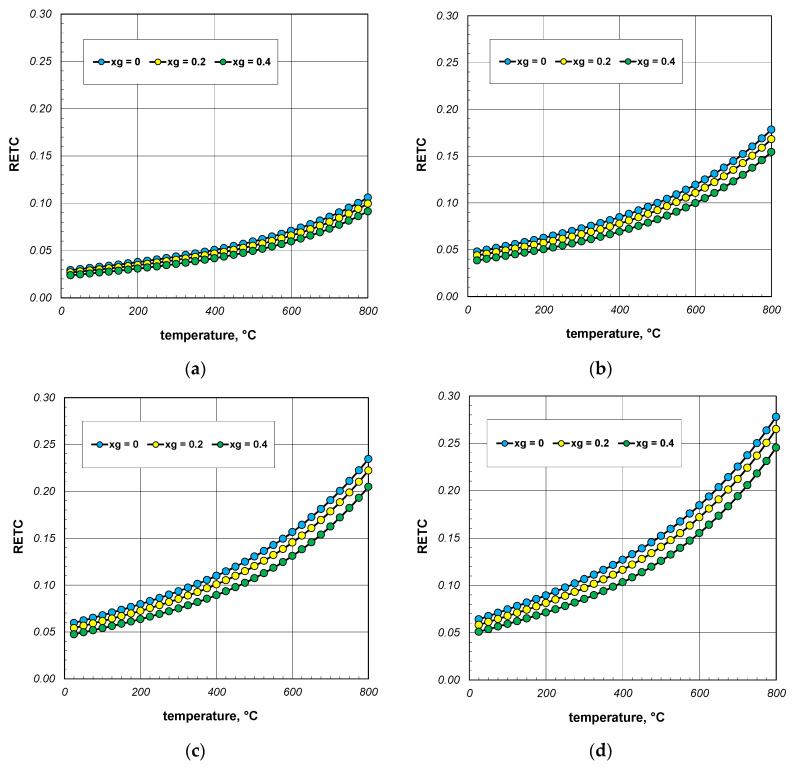
Reduced effective thermal conductivity of the bar bundle depending on the temperature and porosity: (**a**) 10 mm bars, (**b**) 20 mm bars, (**c**) 30 mm bars, and (**d**) 40 mm bars.

Graphs with the results of the RETC parameter calculations are presented in Figure 7. The discussed quantity increases significantly as a function of temperature. A similar effect, but with much less intensity, causes an increase in the bar diameter. For all cases considered, RETC ranges from 0.03 to 0.28, with an average value of approximately 0.12. These results indicate that the intensity of heat flow in the bar bundle is approximately an order of magnitude smaller than that of conduction in the bars. This shows how different the physical process of heating a bundle is from that of heating a solid steel charge.

**Figure 8:**
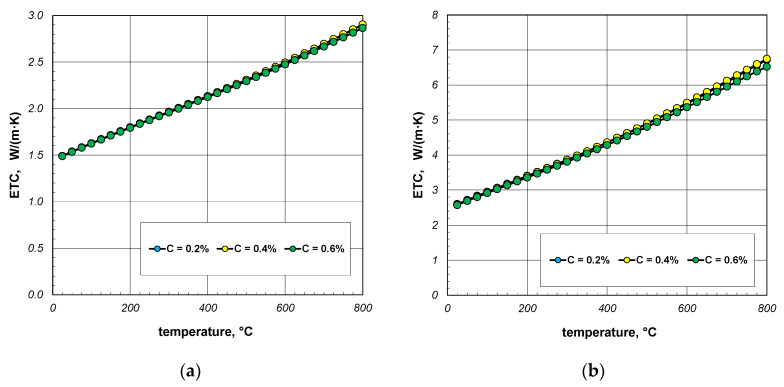
ETC of the 10 mm bar bundle depending on temperature and bar thermal conductivity: (**a**) bundle porosity 0.09 (*x*_g_ = 0) and (**b**) bundle porosity 0.21 (*x*_g_ = 0.4).

**Figure 9:**
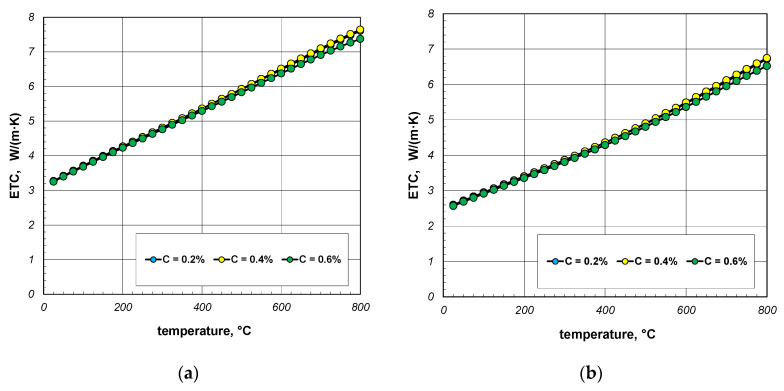
ETC of the 40 mm bar bundle depending on temperature and bar thermal conductivity: (**a**) bundle porosity 0.1 (*x*_g_ = 0) and (**b**) bundle porosity 0.214 (*x*_g_ = 0.4).

The next stage of the analysis was to investigate the impact of the thermal conductivity of steel on the ETC of the bundle. Calculations were made for three steels with a carbon content of 0.2%, 0.4%, and 0.6%. The thermal conductivity of these steels was calculated using Equation (31). The values of the *k_s_* parameter calculated for selected temperatures are listed in Table 4. The results of ETC calculations for a bundle of 10 mm bars and a porosity of 0.09 and 0.21 are presented in Figure 8. As can be seen, the thermal conductivity of the bars has a negligible impact on the ETC value. Similar results were observed for the 40 mm bar bundle, which is shown in Figure 9.

**Figure 10:**
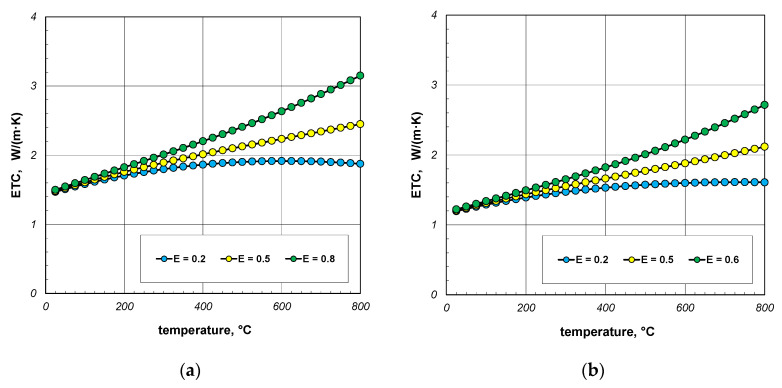
ETC of the 10 mm bar bundle depending on temperature and bar emissivity: (**a**) bundle porosity 0.09 (*x*_g_ = 0) and (**b**) bundle porosity 0.21 (*x*_g_ = 0.4).

**Figure 11:**
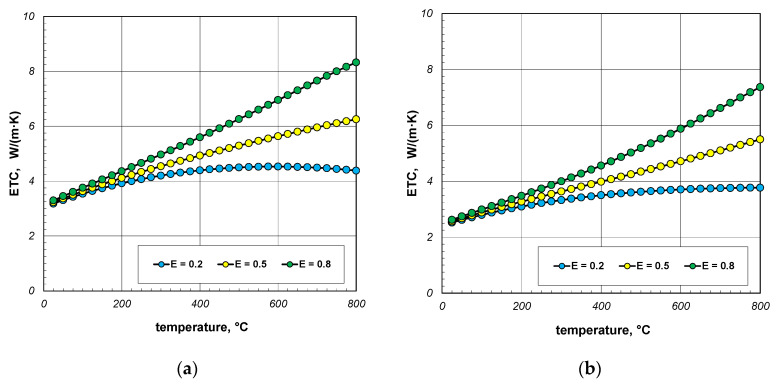
ETC of the 40 mm bar bundle depending on temperature and bar emissivity: (**a**) bundle porosity 0.09 (*x*_g_ = 0) and (**b**) bundle porosity 0.21 (*x*_g_ = 0.4).

As indicated earlier, the phenomenon of thermal radiation is largely responsible for the increase in ETC as a function of temperature. The intensity of this heat transfer mechanism depends on the state of the bar surface, which is defined in the computational model by emissivity. Depending on the surface quality, the emissivity of steel varies within wide limits [60,61]. Heat-treated bars may also have different surface conditions. Therefore, in the next stage of the research, it was decided to check how the emissivity of the bars affects the ETC of the bundle. Calculations were performed for three values of *ε_b_*: 0.2, 0.5, and 0.8. The calculation results obtained for 10 mm and 40 mm bars are shown in Figure 10 and Figure 11. For each of the four bundle geometries considered, it can be seen that after exceeding the temperature of 200 °C, the ETC value strongly depends on the bars’ emissivity. These results indicate that in order to correctly calculate the ETC parameter, it is necessary to accurately identify the bars’ emissivity. An incorrect determination of the *ε_b_* value will result in significant distortion of the calculation results.

**Figure 12:**
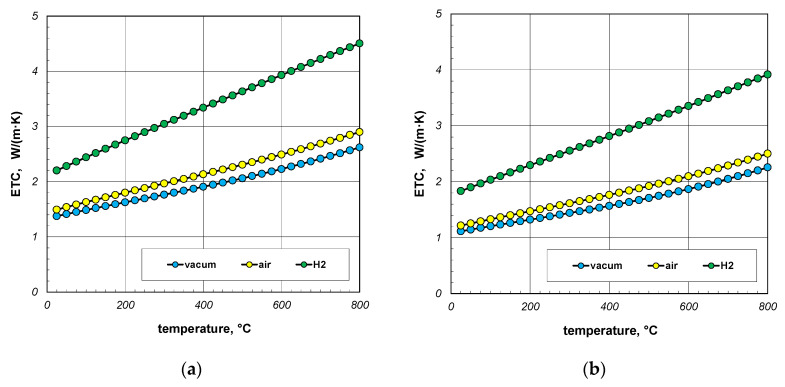
ETC of the 10 mm bar bundle depending on temperature and type of atmosphere: (**a**) bundle porosity 0.09 (*x*_g_ = 0) and (**b**) bundle porosity 0.21 (*x*_g_ = 0.4).

**Figure 13:**
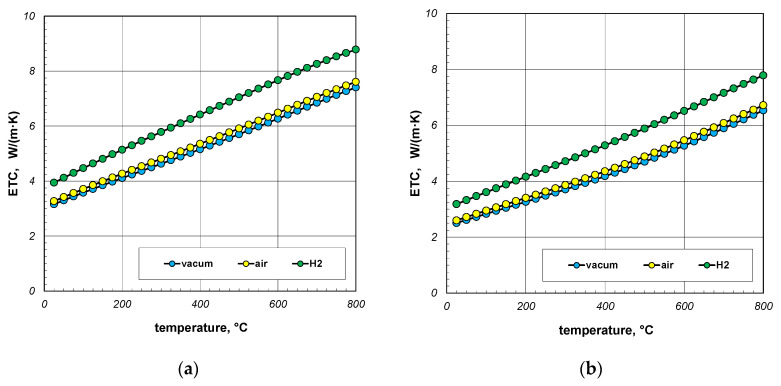
ETC of the 40 mm bar bundle depending on temperature and type of atmosphere: (**a**) bundle porosity 0.09 (*x*_g_ = 0) and (**b**) bundle porosity 0.21 (*x*_g_ = 0.4).

The next point of the analysis was to check how the type of gas used during heating affects the ETC value. Most often, heat treatment of steel products is carried out without the use of a protective atmosphere, i.e., in atmospheric air. However, in many cases, technological requirements force the use of protective atmospheres. As mentioned earlier, hydrogen is often used as a protective gas in the heat treatment of steel products. In special cases, heat treatment is carried out in vacuum furnaces [62,63]. The use of a vacuum has a number of advantages, including eliminating surface oxidation of the heated elements [64]. Calculations in this stage were performed for three cases: vacuum, air, and hydrogen. In the first situation, conduction in the gas phase was omitted in the calculations. However, in the third case, the thermal conductivity of the gas was calculated using Equation (33). The calculation results, taking into account the type of gas obtained for 10 mm and 40 mm bar bundles, are presented in Figure 12 and Figure 13.

The calculations performed show that there is no major difference between vacuum and air. Whereas the use of hydrogen results in a significant increase in the ETC value. For a 10 mm bar bundle, replacing air with hydrogen increases the ETC value by approximately 55% (Figure 12). In the case of a 40 mm bar bundle, this increase is close to 20% (Figure 13). Therefore, when heating a bundle, replacing air with hydrogen causes a significant increase in the intensity of this process. This intensification is stronger the smaller the diameter of the bars.

**Figure 14:**
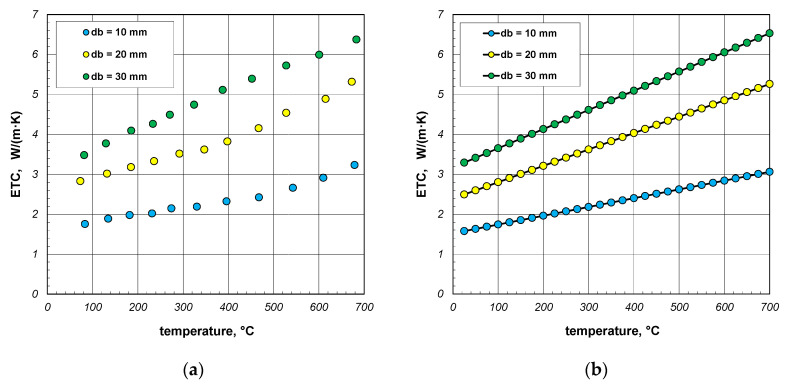
Results of experimental tests on the ETC of the round steel bar bundles [34]: (**a**) measurement data and (**b**) values smoothed using linear regression equations.

The last part of the analysis presented in this article is to evaluate the accuracy of the results generated by the proposed model. For this purpose, the results of experimental tests on the ETC of bundles of round steel bars, which were described in [41], were used. Measurement results obtained for bar bundles 10, 20, and 30 mm are shown in Figure 14a. For the purposes of further analysis, these values were approximated with linear regression functions. For individual samples, these functions take the following forms:

(36) $$ETC_{10}=0.0022t+1.52,$$

(37) $$ETC_{20}=0.0041t+2.39,$$

(38) $$ETC_{30}=0.0048t+3.17.$$

The values of the coefficient of determination *R*^2^ for these functions are within the range of 0.962–0.997. Values close to unity indicate that the selected equations are well aligned with the measurement results. The ETC values smoothed using Equations (36)–(38) are shown in Figure 14b.

**Figure 15:**
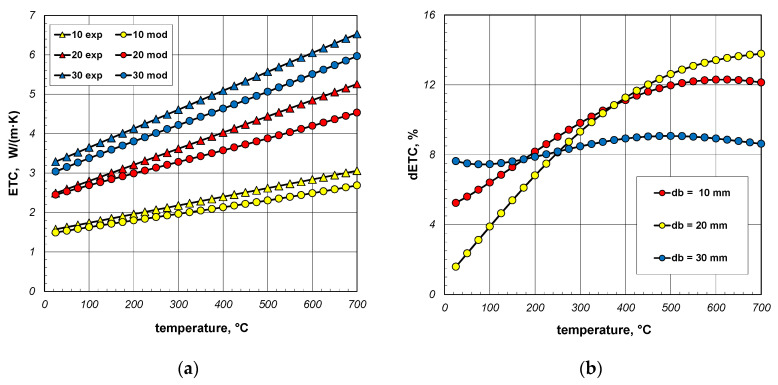
Comparison of measured and model values of ETC (**a**) and changes in the dETC parameter (**b**); results obtained for constant emissivity (*ε_b_* = 0.7).

The results of model calculations and smoothed measurement data are compared in Figure 15a. The samples used during experiments were made from S235JRH steel-grade bars. The maximum carbon content in this steel grade is 0.2% [65]. For this reason, this carbon content was assumed in the model calculations when determining the *k_s_* value (Equation (31)). It was also assumed that the bar’s emissivity is 0.7. As can be seen, for all bundles the measured values are higher than the model values, and this discrepancy increases with temperature. In order to quantify this difference, the dETC parameter was used, defined by the equation:

(39) $$dETC=\frac{ETC_{me}-ETC_{mo}}{ETC_{me}}\cdot 100\%,$$

where ETC_me_—measured values, ETC_mo_—modeled values.

The dETC values are shown in Figure 15b. In the case of 30 mm bars, this parameter changes only slightly and is approximately 8%. In the case of 10 mm and 20 mm bars, dETC increases strongly as a function of temperature, exceeding 12%. It should be mentioned that the maximum uncertainty of ETC measurements was 4.6% [66]. This means that at higher temperatures (above 400 °C) the modeled values deviate too much from the experimental results. It should be mentioned that in the analyzed case the model values were obtained for a constant bar emissivity of 0.7. As experimental tests have shown, the emissivity of steel bars increases during heating. The variation in emissivity of such bars as a function of temperature can be described by the following equation [53]:

(40) $$\varepsilon_{b}\left(t\right)=0.0002\cdot t+0.64$$

**Figure 16:**
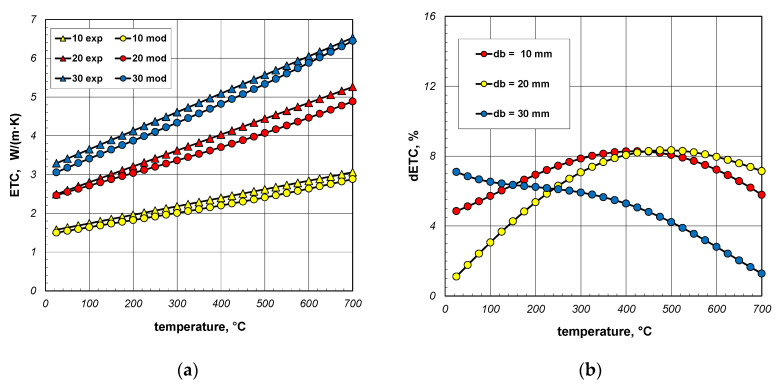
Comparison of measured and model values of ETC (**a**) and changes in the dETC parameter (**b**); results obtained for emissivity described by Equation (40).

Figure 16a again compares the measured and model values of ETC. In this situation, the changes in the bar emissivity as a function of temperature, described by Equation (40), were taken into account in the model calculations. In this situation, the dETC (Figure 16b) values do not exceed 9% and the average value for all bar diameters is approximately 6%. This value is close to the measurement uncertainty of the experimental results. It can, therefore, be concluded that thanks to the change in emissivity, a very good match between the model values and the experimental data was achieved. This confirms the previous conclusion that the key problem for modeling heat transfer in a bar bundle is the selection of the bar’s emissivity.

## 4. Conclusions

The article presents the original model of the effective thermal conductivity of a bundle of round steel bars. This model is based on the analysis of thermal resistances associated with the individual heat transfer mechanisms that occur during the heating of the bar bundle. The heat transfer mechanisms included in the model are: conduction in bars, conduction within the gas, thermal radiation between the bar’s surfaces, and contact conduction across the joints between the adjacent bars. The greatest scientific value of the presented research is the demonstration that thanks to the developed mathematical model, it is possible to analyze a very complex heat transfer phenomenon using relatively simple mathematical relationships. Based on the calculations made, the following conclusions can be drawn:

- ETC of round steel bar bundle changes over the range of 2.2–8.5 W/(m·K);
- ETC rises linearly with temperature;
- ETC increases as a function of the bar diameter—this increase is not linear;
- The bar thermal conductivity does not have much influence on the ETC value;
- The values of ETC range from 0.02 to 0.27 of bar thermal conductivity;
- The key problem for modeling heat transfer in a bar bundle is the selection of the bar’s emissivity;
- Replacing the air with a vacuum does not have a major impact on the bundle heating;
- Replacing air with hydrogen increases the bundle heating intensity from 20% to 55%. The degree of intensification of this process is the greater the smaller the diameter of the bars;
- Changing the bundle porosity in the range of 0.1–0.2 slightly reduces the ETC value.

To sum up the conducted research, it should be emphasized that the influence of contact conduction on the ETC of the bundle was not analyzed. This is a very important problem for the correct modeling of heat transfer in a bar bundle. However, due to the complexity of this issue, its analysis will be the subject of a separate publication.

## Figures and Tables

**Table 1:** Minimum and maximum dimensions of individual elements of a unit cell.

Element	Minimum Dimension	Maximum Dimension
*E*_*I*1_, *E*_*III*1_	*δ*_1_	*δ*_2_
*E_Ig_*, *E_IIIg_*	0	*δ*_3_
*E*_*I*2_, *E*_*III*2_	0	*δ_c_* − *δ*_1_
*E*_*II*1_	*δ*_2_	0.5*d_b_*
*E_IIg_*	*δ*_3_	*δ*_4_

**Table 2:** The thermal conductivities of steel, air, and hydrogen for temperatures 25 °C and 800 °C.

Temperature, °C	Thermal Conductivity, W/(m·K)		
	Steel	Air	Hydrogen
25	51.3	0.026	0.185
800	27.4	0.069	0.495

**Table 3:** Porosity of the bundle depending on the value of the *x_g_* parameter from Equation (3).

*x_g_*	0	0.2	0.4
*φ*	0.09	0.14	0.21

**Table 4:** The values of *k_s_* of steel depending on temperature and carbon content.

Temperature, °C	*k_s_*, W/(m·K)		
	0.2% C	0.4% C	0.6% C
25	51.3	48.0	49.2
100	50.8	47.2	46.4
200	48.3	46.5	43.8
300	44.6	43.8	40.6
400	42.6	41.0	37.6
500	39.2	38.4	34.9
600	35.4	36.0	32.2
700	31.8	31.4	29.1
800	27.4	26.7	24.2

## Data Availability

The original contributions presented in the study are included in the article, further inquiries can be directed to the corresponding author.
